# Burden of disease studies in the WHO European Region—a mapping exercise

**DOI:** 10.1093/eurpub/cky060

**Published:** 2018-04-25

**Authors:** Mark R O’Donovan, Christian Gapp, Claudia Stein

**Affiliations:** 1 School of Public Health, University College Cork, Cork, Ireland; 2 Division of Information, Evidence, Research, and Innovation, WHO Regional Office for Europe, Copenhagen, Denmark

## Abstract

**Background:**

The World Health Organization (WHO) and the Institute for Health Metrics and Evaluation (IHME) have produced numerous global burden of disease (GBD) estimates since the 1990s, using disability-adjusted life-years (DALYs). Here we attempt to identify studies that have either independent DALY estimates or build on the work of WHO and IHME, for the WHO European Region, categorize them by scope of disease analysis and geographic coverage, and briefly compare their methodology (age weighting, discounting and disability weights).

**Methods:**

Google and Google Scholar were used with the search terms ‘DALY’, ‘national burden of disease’, Member State names and researcher’s names, covering all years. Studies were categorized as: ‘specific’ (fewer than five disease categories or just risk factors for a single country), ‘specific, multicountry’ (fewer than five disease categories or just risk factors for more than one country), ‘extensive’ (covering five or more but not all disease categories for one country), ‘full, sub country’ (covering all relevant disease categories for part of one country) and ‘full, country’ (covering all relevant disease categories for one country).

**Results:**

A total of 198 studies were identified: 143 ‘specific’, 26 ‘specific, multicountry’, 7 ‘extensive’, 10 ‘full, sub country’ and 12 ‘full, country’ [England (1), Estonia (2), France (1), Romania (1), Serbia (1), Spain (3), Sweden (2) and Turkey (1)]. About 5 (20%) of the 25 examinable ‘extensive’, ‘full, sub country’ and ‘full, country’ studies calculated DALYs using GBD 2010 methodology.

**Conclusions:**

Independent burden of diseases studies in Europe have been located, and categorized by scope of disease analysis and geographic coverage. Methodological choices varied between independent ‘full, country’ studies.

## Introduction

The burden of disease (BoD) method analyzes the impact of disease upon populations through a combination of mortality and morbidity measures into a single summary statistic of population health.^1^ A summary measure frequently used by the World Health Organization (WHO) and the Institute for Health Metrics and Evaluation (IHME) is the disability-adjusted life-year (DALY).^2^ DALYs quantify disease burden as a health gap; the difference between a hypothetical ideal state of health and wellbeing, and the actual observed health status.^1^ ‘Disability’ in this context refers to any less than ideal health status.^3^

DALYs are calculated through the addition of years of life lost (YLL) and years lost due to disability (YLD) and details of their calculation are provided elsewhere.^3–5^ YLL is an incidence-based measure consisting of the number of deaths multiplied by the standard life expectancy at the age that each death occurred (expected life remaining). YLD is a measure of how many years of healthy life are lost due to time lived in a health status other than optimal health (i.e. disability). Originally, new incident cases were used to maintain consistency with YLL, but more recent methods use prevalence data.^4^^,^^6–8^

YLD calculation relies upon disability weights (DWs) which are socially derived values based on how the majority of people perceive living with a disease or condition for a one year period, where 0 is optimal health and 1 is equivalent to death. These DWs only represent societal preferences about living with a condition and do not represent utility, closeness to death, capabilities or the worth of individuals. DW calculation involves a standardized description of the health states (e.g. EQ-5 D) and valuation methods such as visual analogue scale (VAS), time trade-off (TTO), person trade-off (PTO) or standard gamble (SG). Various DWs have been calculated, e.g. in the global burden of disease (GBD) studies,^9^^,^^10^ the Netherlands^11^ and Estonia.^12^ DWs tend to have reasonably high level of agreement across populations for most conditions, but valuation methods vary significantly.^13^^,^^14^

In early DALY estimates two additional social weights, discounting and age weighting, were widely used. With discounting, less value is applied to future life-years than those lived today, based on the social preference of a healthy year now rather than at a later date. Age weighting intends to adjust for altering levels of dependency with age, as well as possible societal preference for particular ages of life. Ages 0–38 years were favoured by the application of both these social weights.^15^ However, following considerable criticism^15–17^ both age weighting and discounting have been dropped from recent WHO/IHME calculations.^6–8^

As a single number representative of both societal perceptions of morbidity and objective measures of mortality, DALYs are a clear, concise and versatile measure of impact which can be applied to diseases,^18^ risk factors,^19^ interventions^20^ and adverse events.^21^ They can also be easily utilized for risk-benefit analysis^22^ as well as cost-effectiveness studies.^23^^,^^24^ Since DALYs are rooted in societal preferences they can also be considered an important step towards incorporating public opinion in health decision making, and making decisions more representative of population perspectives.

DALYs were initially conceptualized as the health indicator for the first GBD study (GBD 1990) which was directed by Christopher Murray and Alan Lopez under a joint exercise by the WHO and the World Bank.^25^^,^^26^

This original GBD study produced estimates using both ‘adjusted DALYs’ (3% discounting and age weighting) and ‘age-weighted DALYs’ (no discounting, age weighting).^25^ Following this study the WHO^6^^,^^7^ and more recently the IHME^2^ have produced numerous GBD updates. The WHO has been producing various versions of adjusted DALYs, unadjusted DALYs (no discounting, no age weighting) and discounted DALYs (3% discounting, no age weighting) for the years 2000–12, but since the GBD 2010 study unadjusted DALYs have become the established approach.^6–8^

However, in addition to WHO and IHME estimates many other independent studies must have been conducted but their number, scope of disease analysis and methodology remain largely unknown. Previous literature reviews have provided a snapshot of a vast and varied literature with considerable variation in the use of discounting, age weighting, DWs, reference life tables, incidence or prevalence measures and methods handling missing and poorly coded data.^27^

In light of the many potential benefits of measuring BoD and the unknown status and methodology of current independent BoD studies we decided to identify these studies for the WHO European Region, and to map out their extent, scope of disease analysis, geographic coverage and basic methodological choices (in the case of larger BoD studies). This will provide us with an approximation of the current level of BoD usage, capacity, and comparability, hopefully inspire future research, draw attention to the existing literature and promote the use of agreed standardized methodology for local and comparable estimates.

## Methods

Due to the different settings in which BoD research is conducted (by national governments, private sector researchers and academic settings), and the spread of BoD studies across different types of journals and databases, it appeared likely that using conventional search strategies of PubMed, Scopus and similar would underrepresent this literature. To address this concern and increase the sensitivity of the search we moved away from specialized academic databases and instead used Google and Google Scholar as our search engines.

Three main searches were carried out between April and July 2016, as summarized in table 1. The first used the search terms ‘Member State name’, ‘national burden of disease’ and ‘DALY’ for each of the 53 Member States in the Region on both Google and Google Scholar. Unlike the other two searches this first search was mainly concerned with finding full national/country studies and we limited our search engines to only show studies since the year 2000. Since the second GBD study and WHO’s national BoD manual were not published until 2001 we felt that most studies before this time would be methodological discussion papers, and that any independent full national studies in this period should be cited in later publications.^4^^,^^9^^,^^28^

**Table 1:** Summary of study searches conducted (April–July 2016)

No.	Search engine(s)	Search terms	Restriction(s)	Search numbers
1	Google and Google Scholar	‘Member State name’, ‘national burden of disease’, ‘DALY’	Since year 2000	106 searches with 72–538 hits each
2	Google	‘burden of disease’, ‘Member State name’	Default ‘omit similar results’ enabled	53 searches with 142–277 hits each
3a	Google Scholar	‘DALY’, author: ‘full name’	None	>600 researchers 0–104 hits each
3b	Google Scholar	‘DALY’, author: ‘surname’	Only first 100 search hits where >100 hits	>100 researchers 0–6790 hits each

The second search used the search terms ‘burden of disease’ and ‘Member State name’ on Google. The third search used the names of researchers affiliated with BoD research or methodology, using the search terms ‘DALY’ and author: ‘full name’ or ‘DALY’ and author: ‘surname’. These names were obtained via personal communication or from studies found within all three searches.

In addition, reference checks were performed on all accessible eligible studies. These checks identified many additional publications that were eligible for inclusion in this review and these are all included under the applicable search in our results. Reference checks were especially helpful in locating earlier series of burden-calculating reports and poorly indexed studies.

### Inclusion criteria for studies

Based on this review’s inclusion criteria eligible studies were identified from the above search hits by reading abstracts or full texts where available. To be eligible for inclusion in this review, publications had to satisfy the following inclusion criteria. The study:

- uses the DALY metric, providing DALY estimates for a population;
- includes a geographic area within the WHO European Region;
- is the original publication of the estimates (or the earliest located); and
- builds on or modifies WHO and IHME estimates, or is independent research that does not include WHO or IHME estimates.

Publications with original BoD estimates for any part of the European Region were eligible for inclusion; these included Global studies,^18^ as well as cost-effectiveness studies that calculated DALY rates for a European geographic area as part of their analysis.^23^^,^^24^ Studies that did not meet one or more of the above criteria were not included in this review.

### Classification of studies

A first screening of the studies revealed main differences in the scope of diseases and geographic coverage. Therefore, in a second step, studies in this review are classified into five groups based on the scope of disease analysis and their geographic coverage as follows:

- ‘specific’ (covering fewer than five disease categories or just risk factors for all/part of one European country);
- ‘specific, multicountry’ (covering fewer than five disease categories or just risk factors for all/parts of more than one European country);
- ‘extensive’ (covering five or more but not all disease categories for all/part of one European country);
- ‘full, sub country’ (covering all relevant disease categories and representative of part of one European country, e.g. a region, city or population subgroup); and
- ‘full, country’ (covering all relevant disease categories and representative of the whole of one European country).

The term ‘country’ here can refer to just one constituent country or territory in the case of larger sovereign countries. This means, e.g. that a study in Denmark would not need to include the other two constituent territories (Greenland and the Faroe Islands) to be categorized as covering a whole country and that a full study in England, Wales, Scotland, or Northern Ireland would be categorized as a ‘full, country study’. This decision was made since research tends to take place at the constituent country or territory level; e.g. in UK responsibility for health and public health has been devolved to its constituent countries since 1998.^29^

The term ‘disease category’ here refers to any of the 23 main cause categories utilized in recent WHO Global Health Estimates publications.^6^^,^^7^ In the case of research examining just risk factors (e.g. BoD caused by environmental exposures) we have classified them as ‘specific’ studies since they focus on a specific category of risk factors. In doing so we are categorizing studies in terms of completeness of disease/risk coverage, and are not referring to the quality or quantity of work involved.

### Analysis of methodological choices

Finally we conducted a brief methodological analysis of ‘extensive’, ‘full, sub country’ and ‘full, country’ studies looking at the use of age weighting, discounting, and DWs (see Supplementary content 1). They were classified into the four following categories:

- ‘adjusted DALYs’ (3% discounting and age weighting);
- ‘discounted DALYs’ (3% discounting, no age weighting);
- ‘age-weighted DALYs’ (no discounting, age weighting); and
- ‘unadjusted DALYs’ (no discounting, no age weighting).

DWs were classed as either GBD DWs (including modified and updated), or national DWs (Dutch and Estonian).

## Results

### Number of studies identified

From the three searches outlined in table 1, including reference checks, a total of 198 studies were identified that were eligible for inclusion in this review: 63 from search 1, 51 from search 2 and 84 from search 3.

### Study types

About 85% (169/198) of studies looked at a small range of diseases or just risk factors; of these, 143 involved a population from a single country (‘specific’) and 26 spanned 2 or more Member States (‘specific, multicountry’). No multicountry studies looked at more than two disease categories or risk factors.

The remaining 15% (29/198) of studies looked at a large number of or all main diseases; of these, 7 looked at 5 or more disease categories (‘extensive’) and 22 covered all disease categories. Of the 22 looking at all disease categories, 12 covered a full country population (‘full, country’) and 10 were for a population below country representation (‘full, sub country’). A geographic breakdown of the numbers and types of studies is shown in figure 2.

### Years of publication

**Figure 1:**
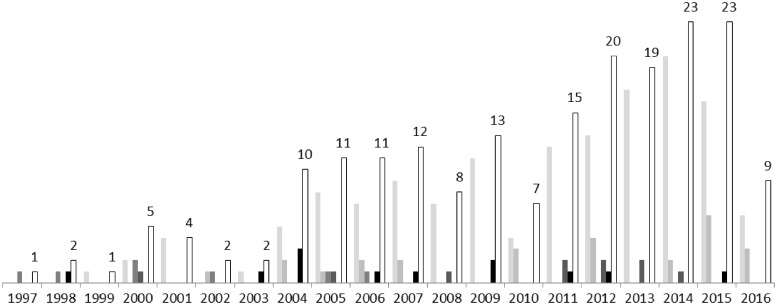
Number of burden of disease studies published each year. Colours of each bar provide a breakdown of the types of studies conducted each year as follows: light grey (leftmost) bar—specific studies; dark grey (second from left) bar—specific, multicountry studies; very dark grey (third from left) bar—extensive studies; darkest grey (fourth from left) bar—full, sub country studies; black (fifth from left) bar—full, country studies; numbered white bar with a black outline (rightmost)—total number of BoD studies ^a^Please note that the online searches for this review were conducted between April and July 2016.

As seen in figure 1 the earliest study located was published in 1997, with a steady increase in the number of studies published each year up to the latest full year of 2015. This is mainly due to an increase in the number of ‘specific’ and ‘specific, multicountry’ studies. (Note: the results for 2016 are not representative of the full year.)

### Geographic coverage

Of the 26 specific, multicountry studies, 17 studied all/most of the WHO European Region (>30 Member States) and 12 of these were global studies. The other nine studied between two and eight Member States.

**Figure 2:**
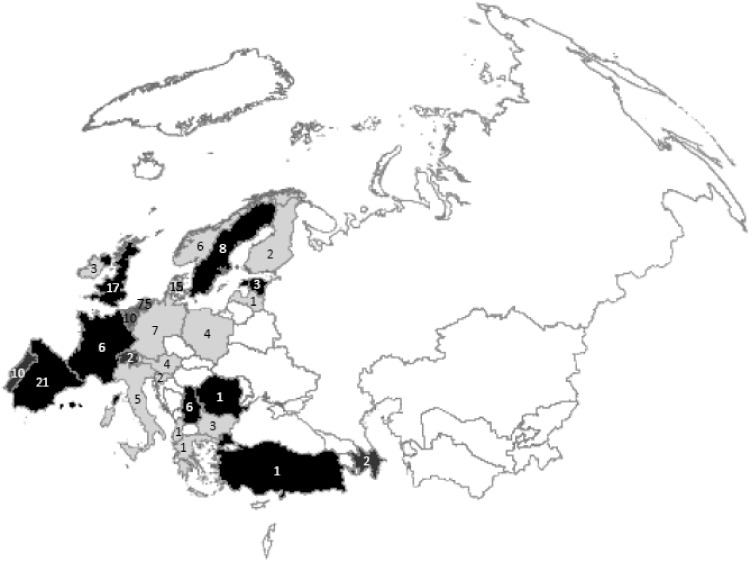
Map of the WHO European Region showing the total number of burden of disease studies conducted for each Member State and the most comprehensive type of study for each. Colours illustrate the most comprehensive type of study for each Member State as follows: light grey shading with black text—specific studies (including multicountry^a^); dark grey shading with black text—extensive study; dark grey shading with white text—full, sub country study; black shading with white text—full, country study^b^^a^Please note that specific multicountry studies that cover over half the Member States (*N* =17) are not included on this map. ^b^Please note that the full, country study for UK only covers England, not the whole of UK

Excluding the 17 specific, multicountry studies that cover all/most of the Region, publications were still found for about half of Member States in the Region: 26 of 53. As shown in figure 2 the largest number was in the Netherlands (75), followed by Spain (21), UK (17), Denmark (15), Belgium (10), Portugal (10), Sweden (8), Germany (7), France (6), Norway (6), Serbia (6), Italy (5), Austria (4), Poland (4), Bulgaria (3), Estonia (3), Ireland (3), Azerbaijan (2), Finland (2), Slovenia (2), Switzerland (2), Albania (1), Greece (1), Latvia (1), Romania (1) and Turkey (1). [Note: the sum of these studies adds up to more than the total number of studies (*N* = 198) owing to inclusion of the nine smaller ‘specific, multicountry’ studies under multiple Member States.]

Full, country studies were found for England (1), Estonia (2), France (1), Romania (1), Serbia (1), Spain (3), Sweden (2) and Turkey (1).

### Methodological choices

Of the 29 ‘extensive’, ‘full, sub country’ and ‘full, country’ studies 25 had enough available data to examine their methodology (two unavailable online, two unexaminable owing to language barriers). Including published, partly-published and unpublished analysis some of the 25 studies include more than one of the DALY categories outlined in our methods section (adjusted DALYs, discounted DALYs, age-weighted DALYs and unadjusted DALYs). For these DALY categories, 20 studies (80%) include 1, 4 studies (16%) include 2 and 1 study (4%) includes 3 DALY categories (31 DALY category estimates in total). Including studies under all relevant categories: 17 studies (68%) calculated adjusted DALYs, 3 studies (12%) calculated discounted DALYs, 2 studies (8%) calculated age-weighted DALYs and 9 studies (36%) calculated unadjusted DALYs.

In total five studies (20%) used national DWs, with two using Estonian weights and three using Dutch weights. Four of these used unadjusted DALYs, and all five were published before the GBD 2010 study when this approach became the norm for GBD estimatimation.^6^^,^^8^

In total five (20%) used unadjusted DALYs with GBD DWs and four of these were published before the GBD 2010 study.

## Discussion

This paper has mapped the extent, scope of disease analysis and geographic coverage of BoD research for the WHO European Region until mid-2016. Since this date additional studies may have been published, one example being the comprehensive Scottish BoD study.^30^

We were surprised to find nearly 200 BoD studies for the Region, but these were mainly specific in focus, examining a limited number of diseases or just risk factors. About 85% of publications used DALYs for these specific research topics. Nevertheless, this illustrates that BoD methods are widely used and valued throughout the Region by numerous researchers. Most of the publications examining all disease categories were conducted in Spain, with a total of seven: three full, country studies and four full, sub country studies. Others, such as a full, country study in France or full, sub country studies in Azerbaijan and Portugal, remain rarely cited.

The majority of BoD publications comes from the Netherlands, where the methodology is highly integrated into national disease reporting in many different specific areas.^31^^,^^32^ However, these include only three extensive studies and no full studies. This approach of using BoD analysis in a fragmented topic-specific manner (seen in the Netherlands and across the European Region) is generally not ideal, as it tends to overestimate the impact of studied conditions and excludes other important diseases and risk factors. Only full BoD studies avoid large over-attributions of burden to specific individual conditions and give more accurate, balanced estimates.^33^ In our view, full, country and full, sub country studies provide the most robust data for policy.

Our analysis shows that BoD studies were carried out primarily in the western part of the Region (see figure 2) and highlights areas without independent BoD publications. We hope that these gaps in the literature will soon be addressed as they would allow for the use of local data for the estimation of BoD without the need to ‘borrow strength’ from data of other countries.

While this review makes important observations it does have a number of limitations. First, the terminology of search terms and their combinations was intentionally narrow—‘burden of disease’ was used but ‘disease burden’ was not. A preliminary test showed that the latter phrase was too unspecific and yielded too many studies that in the end were not dealing with BoD using DALYs. Longer, more robust, search terms could have been used, as well as search engines other than Google and Google Scholar. However, given the large number of searches and reference checks conducted we consider these limitations minor. A second and more serious limitation was that the search was only conducted in the English language and this language bias may explain the Western European concentration of publications observed. Finally, not all research is published online. This was in fact the case with two of the full, country studies in this review which were only cited and described in other available sources. The extent of this unpublished BoD research remains unknown.

Given our findings, the next step could be to analyze methodological differences in more detail, with the aim of harmonizing methods in Europe and making BoD estimates more accessible to policy-makers. Recent collaborative efforts between WHO and IHME have already made progress towards this aim,^34^ including establishing the European Burden of Disease Network (EBoDN) in September 2016.^35^^,^^36^ This is the first health information network of its kind and operates under the umbrella of the WHO European Health Information Initiative,^37^ which guides all health information activities of the WHO Regional Office for Europe.

Our analysis highlights that more countries should embrace the BoD approach in order to enhance local and comparable estimates across the Region and inform national health policy-makers. The variations in methodology in the studies identified confirms the need for the establishment of the EBoDN, which aims to harmonize BoD methods across the Region and engage all Member States, in particular through the development of a national BoD manual that will guide national researchers to perform such studies at the country level. This collaboration in BoD analysis and evidence-informed research is paving the way for better policy-making in the interest of public health and wellbeing.

## Supplementary data

Supplementary data are available at *EURPUB* online.

## Supplementary Material

Supplementary Data
