# Supplementary material for: Burden of disease studies in the WHO European Region—a mapping exercise
Source: Eur J Public Health. 2018 Apr 25;28(4):773–8. doi: 10.1093/eurpub/cky060 (PMC6093319; doi:10.1093/eurpub/cky060)
Supplement: Supplementary Data [file cky060_supp.zip › cky060-suppl_data/ejph-2017-09-om-0709-File004.doc]

**Full, country studies (N = 12)**

**Full, country burden of disease studies conducted throughout the WHO European Region, with information on the population (country) studied, the year of publication of the study, whether social weights (age weighting and discounting) were used, what disability weights were used, and basic details of the analyses.**

**- Bullet points under the ‘Population(s)’ and ‘Age weighting & discounting (3%)’ sections refer to additional analyses.**

**Choices that vary from the methodology used in the GBD 2010 are shaded in grey.**

| **Researchers** | **Population(s)** | **Year published** | **Age weighting & Discounting (3%)** | **Disability weights** | **Details of analysis** |
| --- | --- | --- | --- | --- | --- |
| Peterson et al.  [citation only] | Sweden | 1998 | Unknown | Unknown | Google Scholar citation only  Referenced elsewhere as a full study |
| Atanasković-Marković et al. | Serbia | 2003 | Yes  - YLLs without age weighting  - No discounting (unpublished) | GBD | Reference year 2000  135 diseases  7 risk factors |
| Ersoy et al. | Turkey  - 5 sub-regions,  - urban/rural | 2004 | Yes  - No discounting  - Discounting 6%  - Without age weighting | GBD | Reference year 2000  8 risk factors |
| Galan  [mentions study in a Health Systems handbook] | Romania | 2004 | Unknown | Unknown | Results only  Reference year 1998  By Institute of Public Health Bucharest |
| Lai et al. | Estonia | 2004 | No | National (Estonian) | Reference years 2000, 2001, 2002  168 diseases |
| Allebeck et al. | Sweden | 2006 | No  - Yes | GBD | Reference years 2002/2003  136 diseases  26 risk factors |
| Lapostolle et al. | France | 2007 | Yes  - No | GBD | Reference years 2001–2002 |
| Catalá-López et al. | Spain | 2009 | Yes | GBD | Reference year 2006  Comparison with health care spending |
| Lai et al. | Estonia | 2009 | No | National (Estonian) | Reference year 2002  168 diseases |
| Catalá-López et al. [update of Catalá-López et al., 2009] | Spain | 2011 | Yes | GBD | Reference year 2006 [update]  Comparison with health care spending |
| Gènova-Maleras et al. | Spain | 2012 | Yes | GBD | Reference year 2008 |
| Newton et al. | England  - 9 subregions | 2015 | No | GBD | Reference years 1990–2013  306 diseases  79 risk factors |

**Full, sub country studies (N = 10)**

**Full, sub country burden of disease studies conducted throughout the WHO European Region, with information on the population studied, the year of publication of the study, whether social weights (age weighting and discounting) were used, what disability weights were used, and basic details of the analyses.**

**- Bullet points under the ‘Age weighting & discounting (3%)’ section refer to additional analyses.**

**Choices that vary from the methodology used in the GBD 2010 are shaded in grey.**

| **Researchers** | **Population(s)** | **Year published** | **Age weighting & Discounting (3%)** | **Disability weights** | **Details of analysis** |
| --- | --- | --- | --- | --- | --- |
| Schopper et al. | Geneva, Switzerland | 2000 | Yes | GBD | Reference years 1990–1994 |
| Granados et al. | Paris, France | 2005 | Yes | GBD | Reference year 1999 |
| Dodhia &. Phillips | Two inner London boroughs, England, UK | 2008 | Discounting 3%  - No (unpublished) | GBD | Reference year 2005 |
| Gènova-Maleras et al. | Over 60-year-olds, Spain | 2011 | Yes (unclear) | GBD | Reference year 2008 |
| Machado et al. | Northern region of Portugal | 2011 | Yes | GBD | Reference years 2003–2005 |
| Farahbakhsh et al. | Women, Azerbaijan | 2012 | Unknown | GBD | Probably all GBD diseases  Reference year 2007 |
| Hakimi et al. | East Azerbaijan | 2012 | Yes (unclear) | GBD | Probably all GBD diseases  Reference year 2007 |
| Catalá-López et al. | Valencia, Spain | 2013 | Yes | GBD | Reference year 2008 |
| Catalá-López et al. | 15–29-year-olds, Spain | 2013 | Yes | GBD | Reference year 2008 |
| Andreu & Ricart | Catalonia, Spain | 2014 | Yes (unclear) | GBD | Reference years 2005–2010  123 diseases |

**Extensive studies (N = 7)**

**Extensive burden of disease studies conducted throughout the WHO European Region, with information on the population studied, the year of publication of the study, whether social weights (age weighting and discounting) were used, what disability weights were used, and basic details of the analyses.**

**Choices that vary from the methodology used in the GBD 2010 are shaded in grey.**

| **Researchers** | **Population(s)** | **Year published** | **Age weighting & Discounting (3%)** | **Disability Weights** | **Details of analysis** |
| --- | --- | --- | --- | --- | --- |
| Bowie et al. | South-west region, England, UK | 1997 | Yes | GBD | Reference year 1994  Own diseases groups |
| Melse & Kramers | Netherlands | 1998 | No | National (Dutch) | Reference year 1997  52 diseases |
| Hollinghurst et al. | South-west region, England, UK | 2000 | Discounting 3% | GBD (modified) | Reference years 1990–1995  Own diseases groups |
| Melse & Kramers | Netherlands | 2000 | No | National (Dutch) | Reference year 1994  48 diseases |
| Baert et al. | Flanders, Belgium | 2002 | Yes | National (Dutch) | Reference year 1997  44 diseases |
| Ljung et al. | 3 age groups (15–44, 45–64, 65–84), Sweden | 2005 | No | GBD | Reference years 1988–1995  18 disease categories |
| de Hollander et al. | Netherlands | 2006 | Unknown | Unknown | Reference year probably 2003  7 disease categories |
